# Expanded graphite embedded with aluminum nanoparticles as superior thermal conductivity anodes for high-performance lithium-ion batteries

**DOI:** 10.1038/srep33833

**Published:** 2016-09-27

**Authors:** Tingkai Zhao, Shengfei She, Xianglin Ji, Xinai Guo, Wenbo Jin, Ruoxing Zhu, Alei Dang, Hao Li, Tiehu Li, Bingqing Wei

**Affiliations:** 1 School of Materials Science and Engineering, Northwestern Polytechnical University, Xi’an 710072, China; 2 Department of Mechanical Engineering, University of Delaware, Newark DE 19716, United States

## Abstract

The development of high capacity and long-life lithium-ion batteries is a long-term pursuing and under a close scrutiny. Most of the researches have been focused on exploring electrode materials and structures with high store capability of lithium ions and at the same time with a good electrical conductivity. Thermal conductivity of an electrode material will also have significant impacts on boosting battery capacity and prolonging battery lifetime, which is, however, underestimated. Here, we present the development of an expanded graphite embedded with Al metal nanoparticles (EG-MNPs-Al) synthesized by an oxidation-expansion process. The synthesized EG-MNPs-Al material exhibited a typical hierarchical structure with embedded Al metal nanoparticles into the interspaces of expanded graphite. The parallel thermal conductivity was up to 11.6 W·m^−1^·K^−1^ with a bulk density of 453 kg·m^−3^ at room temperature, a 150% improvement compared to expanded graphite (4.6 W·m^−1^·K^−1^) owing to the existence of Al metal nanoparticles. The first reversible capacity of EG-MNPs-Al as anode material for lithium ion battery was 480 mAh·g^−1^ at a current density of 100 mA·g^−1^, and retained 84% capacity after 300 cycles. The improved cycling stability and system security of lithium ion batteries is attributed to the excellent thermal conductivity of the EG-MNPs-Al anodes.

Due to the increasing interests on the environmentally friendly electrochemical energy conversion and storage devices, lithium ion battery (LIB) is one of the most promising candidates to be rechargeable power supplies for various applications, ranging from portable electronics to low- or zero-emission electric vehicles (EVs) or hybrid EVs (HEVs)^1^,^2^,^3^,^4^,^5^,^6^,^7^,^8^. Although LIBs have many advantages such as high energy and environmental benignity, there are still some challenges to be addressed in practical applications such as safety protection, temperature limitation, and age-related degenerative effects. Obviously, all of these challenges are associated with heat generation inside the batteries. During charge/discharge cycles, the batteries generate significant amount of heat because of various chemical and electrochemical reactions. The accumulated heat contained within the battery’s package is the dominant cause of safety risks such as overheating, combustion, and explosion of the entire battery systems^9^,^10^. Furthermore, excessive heat would speed up the capacity deterioration and thus cause reduction of cycle life^10^. Therefore, heat management is a crucial issue for the research and development of high-performance LIBs.

As the overall performance of LIBs depends intimately on the physical and chemical properties of electrode materials, considerable efforts have been devoted to the research and development of these materials^2^,^3^,^4^,^5^,^11^,^12^. Graphite is the dominant anode material for commercial LIBs owing to its low-cost, safety, and high Coulombic efficiency (CE); however, the limited theoretical capacity (372 mAh·g^−1^) restricts its applications^13^. Various attempts to construct channels and defects on graphite have been carried out so as to enhance the reversible capacity^14^,^15^,^16^. An expansion of graphite interlayer-distance seems to be an appropriate approach to considerably enhance the reversible capacity^17^,^18^,^19^. Expanded graphite (EG), as a graphite-derived material, possesses an analogous long-range-ordered layered structure but with a larger interlayer lattice distance (>0.34 nm)^20^,^21^. Due to its extraordinary properties such as good flexibility, great chemical tolerance, high electrical and thermal conductivities, and outstanding thermal shock resistance, EG has wide-range industrial applications as antistatic coatings, infrared shield materials, thermal transport media, and electronic components^22^,^23^. As for its application in energy conversion and storage fields, many investigations have been carried out on its thermal conductivity and electrochemical properties. M. Bonnissel *et al*.^23^ studied the thermal conductivities of compacted exfoliated graphite samples, and the parallel thermal conductivity reached a maximum value of 9 W·m^−1^·K^−1^ with a bulk density of 400 kg·m^−3^. J. Bodzenta *et al*.^24^ reported that the thermal conductivity value of compressed EG was about 10 W·m^−1^·K^−1^ with an apparent density of 240 kg·m^−3^. In addition, Hyun D. Yoo *et al*.^17^ reported an EG prepared from the heat-treatment of graphite oxide, exhibiting a reversible capacity of 300 mAh·g^−1^ after 20 cycles. Yang *et al*.^18^ prepared expanded mesocarbon microbeads (MCMB) by an intercalation reaction and rapid heating process, and this material presented a reversible capacity of 260 mAh·g^−1^ and good cycling performance with almost 100% capacity retention after 50 cycles. Park *et al*.^19^ prepared various EG materials using an oxidation-expansion process and the optimized anode sample showed an initial reversible capacity of about 400 mAh·g^−1^, but the cycle performance was very poor. However, the investigations concerned both the thermal conductivities and electrochemical properties of EG materials for LIBs have not been reported yet. The synergetic effect of the reversible capacity, cycling stability, and safety issues are very important and still challenging tasks for LIB applications. Therefore, to design a negative electrode material with good thermal conductivity for a high heat-dispersion Li-ion cell is believed to be a promising strategy to overcome these issues^10^,^25^.

In this work, a material based on expanded graphite embedded with Al metal nanoparticles (EG-MNPs-Al) was synthesized via the oxidation-expansion process using natural flake graphite as the raw material and an Al compound as the expanding agent. The EG-MNPs-Al material exhibited a high thermal conductivity and excellent electrochemical performance. The effects of the expanding agents on the properties of the EG-MNPs material play a key role. And an interpretative mechanism has been proposed to illustrate a possible intercalation process. This material as anode materials for LIBs may be helpful to solve the heat-related issues in practical applications.

## Results

**Figure 1:**
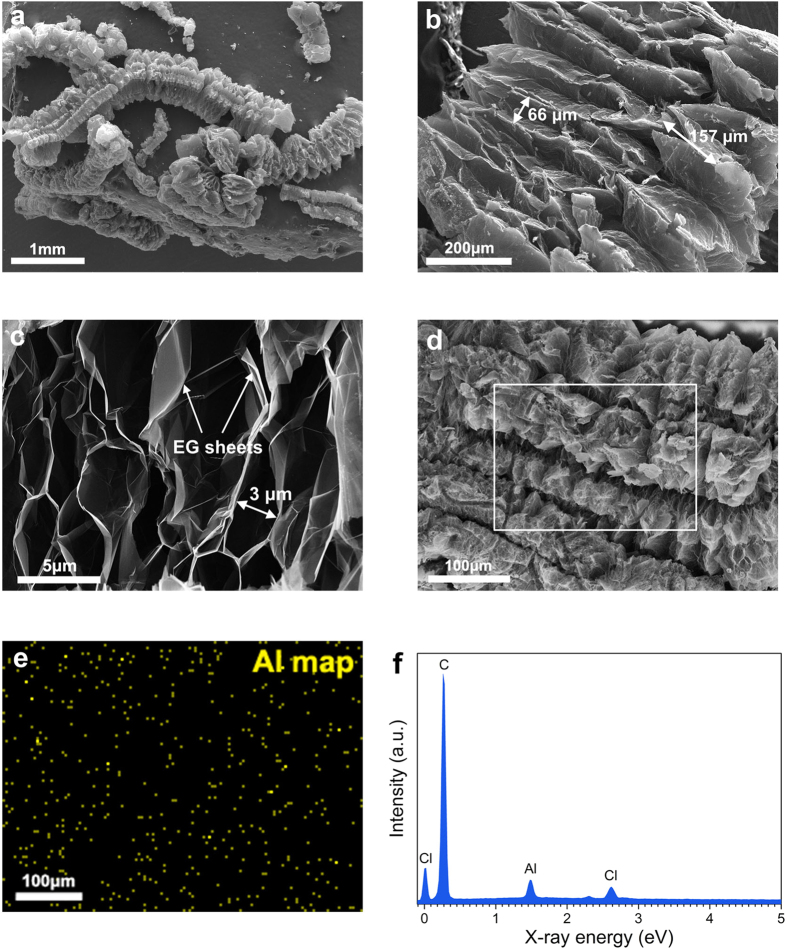
The morphology and microstructure of EG-MNPs-Al. (**a**) SEM image of macroscale EG-MNPs-Al material. (**b**) SEM image of the expanded orientation of EG-MNPs-Al. (**c**) SEM image of the interspaces of EG-MNPs-Al sheets. (**d**) SEM image of worm-shaped EG-MNPs-Al, (**e**) corresponding Al elemental map and (**f**) EDS spectrum of the area outlined by the white square in (**d**).

The SEM images and elemental analysis shown in Fig. 1 characterize the morphology and structure of the EG-MNPs-Al material. Figure 1a,b clearly present that the EG-MNPs-Al has a worm-like structure with more than 100 μm large size sheets, and large amounts of slit-shaped pores between the graphite platelets are clearly visible. Figure 1b also shows the presence and orientation of EG sheets in the resulting product, indicating that the natural flake graphite (NFG) expands along *c*-axis (the direction perpendicular to the graphite layers). Figure 1c exhibits that the EG-MNPs-Al possesses an ideal layer-by-layer structure with an enlarged interlayer spacing and retains an analogous long-range-ordered layered structure^20^,^26^. It can be attributed to the enormous volume increase during the thermal treatment process. Additionally, at a high temperature, the intercalation agents decompose and force the adjacent graphite layers of graphite intercalation compound (GIC) to separate from each other randomly^27^. At high magnifications, a kind of honeycomb-like microstructure consisting of many translucent and wrinkled paper-like graphene sheets are observed in Fig. 1c 28. Due to the discontinuous aggregation of curled EG sheets, there are also abundant pores of different sizes (the width of pores is about 3 μm) ranging from macroscale to nanoscale^26^. Therefore, the interspaces between the EG sheets are easily embedded with small particles and thereby the Al metal nanoparticles could easily insert into the interspaces. The morphology of EG-MNPs-Al shown in Fig. 1d is similar to that of the pure EG, signifying that the Al existing as small nanoparticles is embedded into the interspaces. The Al elemental map (Fig. 1e) and EDS analysis (Fig. 1f) corresponding to the area outlined by the white square in Fig. 1d show a uniform distribution of Al element in the EG-MNPs-Al sample, confirming that the decomposed Al metal nanoparticles are effectively inserted into the framework of the EG host.

**Figure 2:**
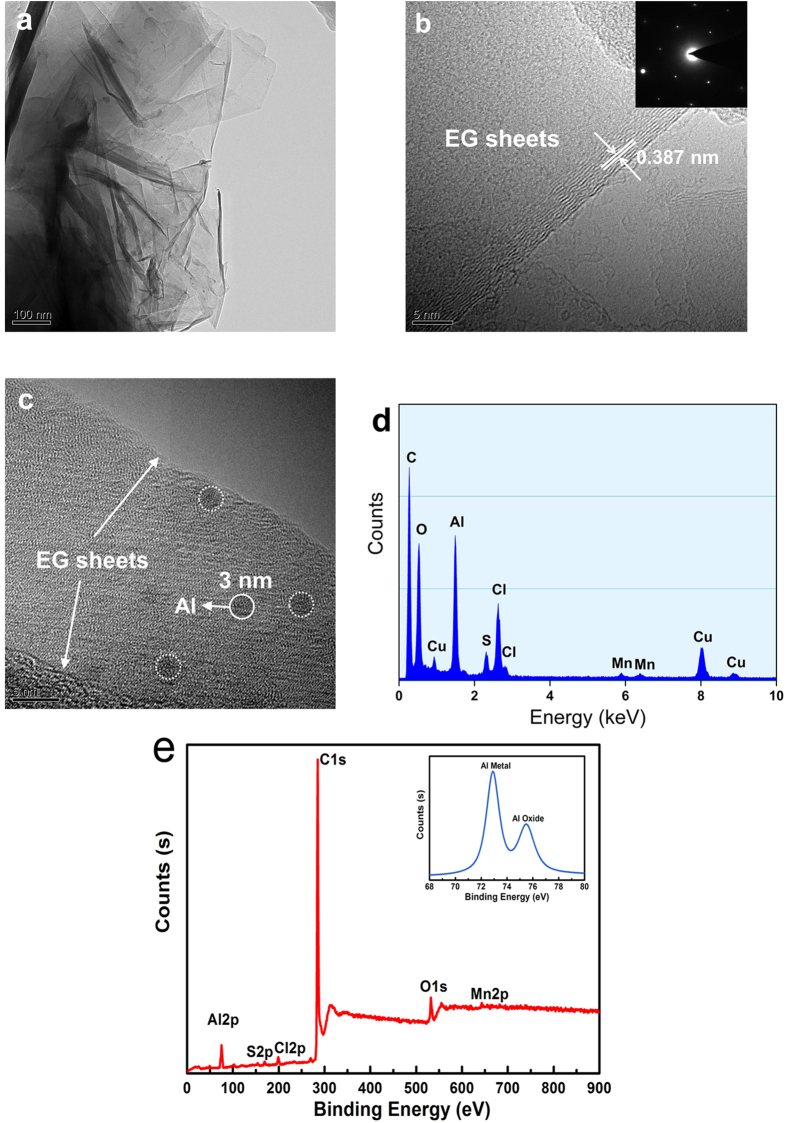
TEM images, EDS and XPS of EG-MNPs-Al. (**a**) TEM image of stacked graphene sheets in EG-MNPs-Al. (**b**) HRTEM image of EG-MNPs-Al where the lattice planes correspond to (002) planes with an enlarged interlayer distance of 0.387 nm. The right-top inset picture is the corresponding selected area electron diffraction (SAED) pattern. (**c**) HRTEM image of a section of EG-MNPs-Al embedded with Al metal nanoparticles, (**d**) corresponding EDS spectrum. (**e**) XPS spectrum of EG-MNPs-Al.

TEM image in Fig. 2a illustrates that the EG-MNPs-Al sample consists of a number of graphene sheets with irregular shapes, and the transparency reveals that the sheets, which resemble a crumpled paper, are composed of a few graphene layers. The disorder-stacking graphene sheets form corrugated and curled structures, and the stacking layers are not commensurate^28^,^29^. The possible reason is that the expanding process parameters can not be efficiently controlled^16^,^26^. Therefore, the disordered aggregation leads to the presence of many nanopores and nanocavities in the scrolled graphene sheets, which are favorable to the intercalation of Li^+^. HRTEM image in Fig. 2b displays that the EG-MNPs-Al possesses the expanded interlayer distances and the long-range channels, which are suitable for lithium ions transport. The average interlayer spacing of (002) plane is measured to be 0.387 nm, which is larger than that of graphite (0.34 nm)^30^, indicating that the distance between graphite layers is enlarged after the oxidation-expansion process because of the insertion of oxygen-containing groups. The right-top inset picture in Fig. 2b is the selected area electron diffraction (SAED) pattern of the corresponding EG-MNPs-Al. The well-defined diffraction spots fully confirm that the crystal-structure of EG-MNPs-Al is hexagonal. The HRTEM image in Fig. 2c illustrates that Al nanoparticles (indicated as a white circle, the diameter is measured to be about 3 nm) are adhered on the interlayers of the EG sheets. Combined with the EDS spectrum (Fig. 2d), it further corroborates that Al metal nanoparticles existed among the EG sheets, in addition to other elements including O (from oxidation process), S (from H_2_SO_4_), Cl (from AlCl_3_), and Mn (from KMnO_4_) formed in the preparation process of EG-MNPs-Al.

From above EDS and TEM results, it has been proved the existence of Al element in the EG interlayers. And then, the chemical state and content of Al element in the EG-MNPs-Al were further investigated using XPS analyzer (Fig. 2e). As can be seen from Fig. 2e, the carbon peak at 285 eV is very sharp and attributed to C1s, and the Al peaks at 72.9 eV and 75.5 eV are attributed to Al2p and Al NtvO*x*, respectively. Furthermore, the specific content can be calculated from the different peaks intensity of EG-MNPs-Al, indicating that it contains 88.7% carbon, 4.6% Al, 4.3% oxygen, 1.1% Cl, 0.8% S, and 0.5% Mn. The profile in the inset (at the right corner of Fig. 2e) exhibits that the chemical state of Al mainly exist in the form of Al metal and oxide, while the Al oxide should be mostly attributed to the surface oxidation of Al nanoparticles in air atmosphere environment. The XPS results on atomic concentrations suggested that the surface elemental composition were consistent with EDS spectrum (Fig. 2d).

**Figure 3:**
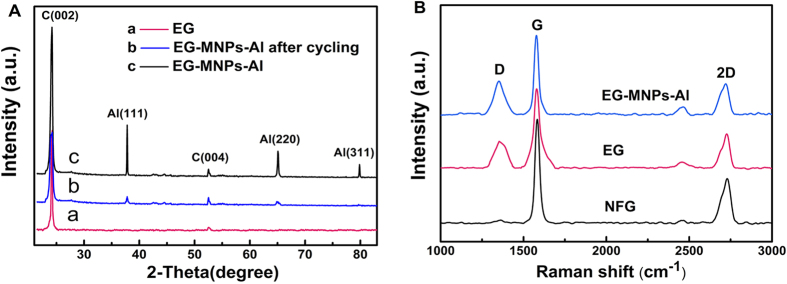
Investigations on the compositions and crystalline structures of the NFG, EG, and EG-MNPs-Al. (**A**) XRD patterns of EG (a), EG-MNPs-Al electrode material after 300 cycles (b) and pristine EG-MNPs-Al (c), (**B**) Raman spectra of NFG, EG and EG-MNPs-Al.

XRD patterns of EG [curve (a)] and EG-MNPs-Al [curve (c)] samples are shown in Fig. 3A. The curve (c) exhibits a sharp (002) characteristic diffraction peak of graphite at 24.2°, and the (002) diffraction peak intensity is stronger than that of the curve (a), and it presents higher crystallinity of EG-MNPs-Al, which is consistent with HRTEM observation. Moreover, the interlayer spacing value *d*_002_ of EG-MNPs-Al is 0.375 nm, larger than that of standard graphite 0.34 nm, indicating that large amounts of graphite flakes have been efficiently expanded and EG-MNPs-Al possesses an enlarged interlayer lattice distance. It is in agreement with the results in Fig. 2b. The (111) reflection reveals the characteristic peak of Al, further indicating the presence of Al metal nanoparticles in EG-MNPs-Al. On the XRD pattern of the EG-MNPs-Al electrode at the end of 300^th^ cycle discharge [curve (b)], the Al characteristic peaks of (111) and (311) planes almost disappear and no additional peaks are formed, indicating that the Al nanoparticles have been consumed during the cycling process and crystalline Li-Al alloys have not been formed. Therefore, it is supposed that the Li_*x*_Al (*x* = 1~2.25) alloys might be an amorphous compound or it might decompose at the end of the discharge^31^.

Raman spectra shown in Fig. 3B characterize the significant structural changes during the oxidation-expansion process from NFG to EG and EG-MNPs-Al. All the Raman spectra exhibit the characteristics of G-band (~1580 cm^−1^), D-band (~1350 cm^−1^) and 2D-band (~2730 cm^−1^), but the D peak of the NFG is not evident in the Raman spectra. As shown in Fig. 3B, the D/G intensity ratio of EG-MNPs-Al (I_D_/I_G_ = 0.414) and EG (I_D_/I_G_ = 0.335) is much larger than that of NFG (I_D_/I_G_ = 0.027), signifying the increase of defects and disorder degree in the EG-MNPs-Al and EG materials, which might be a result of extensive oxidation and rapid thermal exfoliation^17^,^26^,^32^. The increased defects may provide many active sites, and are favorable to the insertion and extraction of Li ions so as to improve the reversible capacity of batteries.

**Figure 4:**
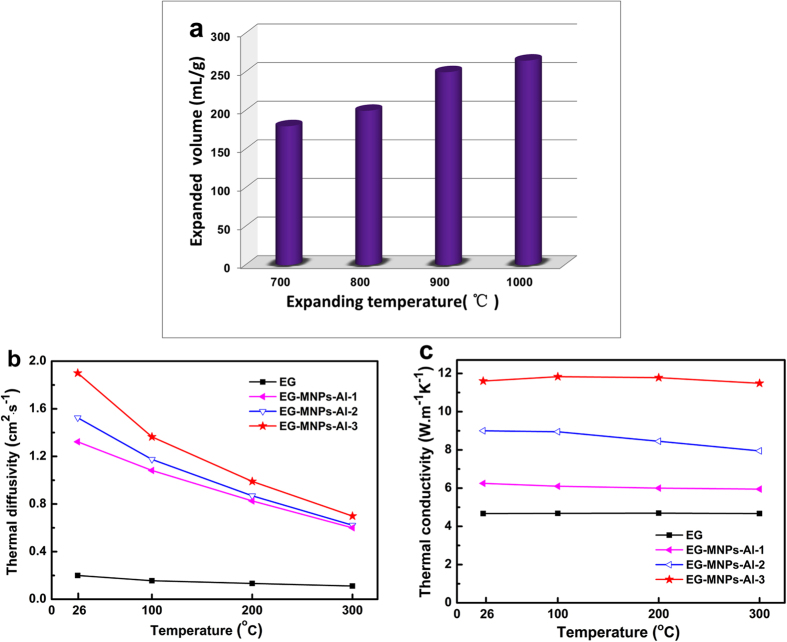
Thermal diffusivity and conductivity of EG and EG-MNPs-Al. (**a**) Expanded volume of EG-MNPs-Al after thermal treatment at 700, 800, 900, and 1000 °C, respectively. (**b**) Thermal diffusivity, and (**c**) thermal conductivity of the samples listed in Table 1 at different temperatures. (**d**) The heat conduction schematic diagram of EG-MNPs-Al material along the direction perpendicular to the graphite layers.

Figure 4a exhibits the influence of expanding temperature on the expanded volume of the EG-MNPs-Al-3 materials. It shows that the expanded volume reaches a maximum value of 265 mL·g^−1^ at 1000 °C and a higher expanding temperature is beneficial to improve the expanded volume. Figure 4b shows the parallel thermal diffusivity for the samples listed in Table 1. It indicates that the parallel thermal diffusivities monotonically decrease with the temperature increase. Most of the heat transfer mainly occurs by phonons^33^. As the temperature goes up, the lattice vibration intensifies and the probability of phonon scattering increases, so the parallel thermal diffusivity decreases. However, there was a noticeable difference in heat conducting capability of the EG and EG-MNPs-Al samples. The parallel thermal diffusivity of EG-MNPs-Al is higher than that of EG and reach a maximum value of 1.89 cm^2^·s^−1^ at room temperature. The insertion of Al metal nanoparticles into the interlayers of EG can be the possible reason for the higher thermal diffusivity. As shown in Fig. 4c, the corresponding parallel thermal conductivities were also calculated using the formula λ = *α* • *ρ* • *c*_*p*_ (λ: thermal conductivity, *α*: thermal diffusivity, *ρ*: density, *c*_p_: specific heat capacity), clearly illustrating the effect of Al adding contents on the parallel thermal conductivity of EG. EG-MNPs-Al materials exhibit a steady improvement in the parallel thermal conductivity compared to the EG materials, and the EG-MNPs-Al-3 attained the highest parallel thermal conductivity value of 11.6 W·m^−1^·K^−1^ with a bulk density of 453 kg·m^−3^ at room temperature, a 150% improvement compared to EG. It is true that the thermal conductivity of Al (~230 W·m^−1^·K^−1^) is much higher than graphite in parallel direction (~10 W·m^−1^·K^−1^) at room temperature, so that the parallel thermal conductivity improvement of EG-MNPs-Al could be attributed to its special structure with Al metal nanoparticles embedded into the interspaces of EG^34^,^35^. Figure 4c also shows that the parallel thermal conductivity of EG-MNPs-Al enhances greatly with the weight ratio of added Al increasing. It further indicates that the Al nanoparticles play a key role in the parallel thermal conductivity improvement^34^,^36^,^37^. In addition, the parallel thermal conductivities of all EG-MNPs-Al samples show little variation with temperature, illustrating an outstanding thermal stability of the EG-MNPs-Al samples^37^.

**Figure 5:**
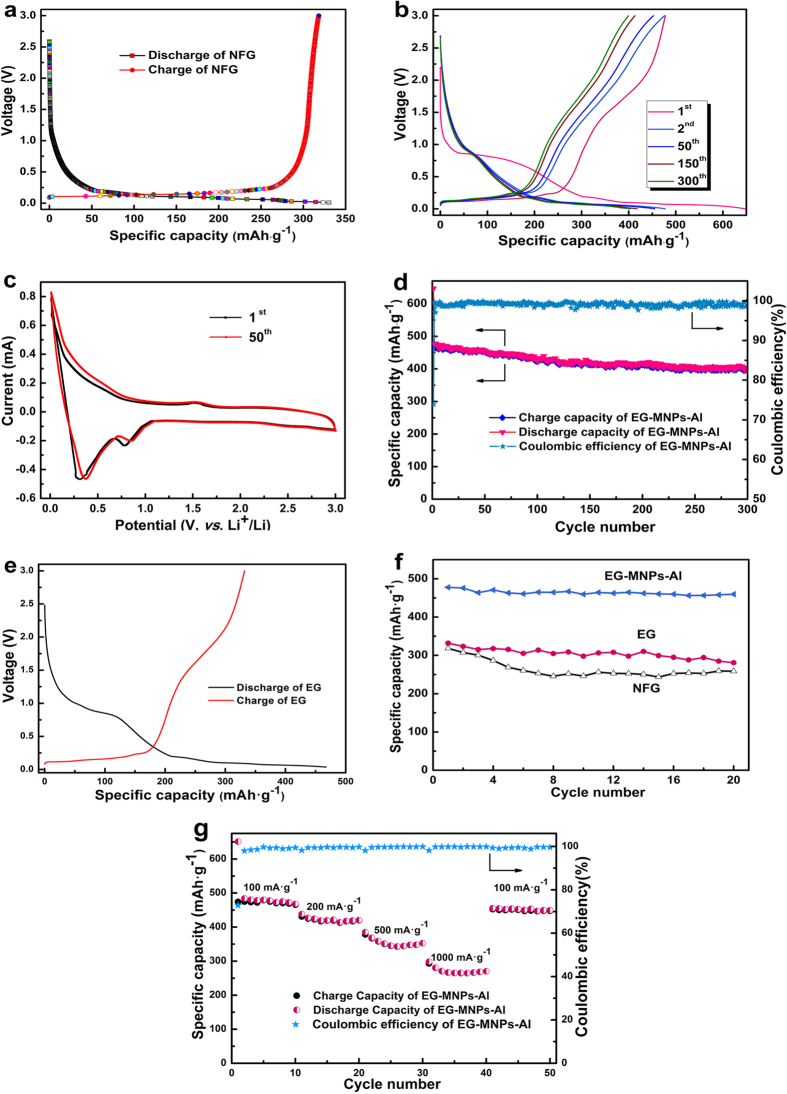
Electrochemical performances of the NFG, EG, and EG-MNPs-Al. (**a**) First charge-discharge profiles of NFG at 100 mA·g^−1^. (**b**) Galvanostatic charge-discharge curves of EG-MNPs-Al electrode at current density of 100 mA·g^−1^. (**c**) Cyclic voltammograms (CVs) of the EG-MNPs-Al anode in a coin cell at a scan rate of 0.1 mV·s^−1^. (**d**) Cycling performance of the EG-MNPs-Al electrode for 300 cycles. (**e**) The initial charge-discharge curves of EG. (**f**) Cycling stability of NFG, EG and EG-MNPs-Al. (**g**) Rate performance of EG-MNPs-Al at different current densities from 100 mA·g^−1^ to 1000 mA·g^−1^.

The galvanostatic charge-discharge profiles of Li-ion batteries were measured to evaluate the electrochemical properties with respect to Li^+^ insertion/extraction of the EG-MNPs-Al-3 material (the next contents use the same samples) at the current density of 100 mA·g^−1^ within a potential window of 0.01~3.0 V *vs*. Li/Li^+^ (Fig. 5). Figure 5a shows the initial charge-discharge profiles of NFG. It presents the typical insertion/extraction properties of highly crystalline graphite electrode materials and a reversible capacity of 318 mAh·g^−1^ was obtained. The charge-discharge curves of the EG-MNPs-Al electrode are presented in Fig. 5b. The initial charge and discharge capacities of EG-MNPs-Al is 647 mAh·g^−1^ and 480 mAh·g^−1^ respectively, which are much higher than those of NFG (Fig. 5a). The particular hierarchical structure of the EG-MNPs-Al material, especially the insertion of Al metal, gives rise to the large reversible capacity. Furthermore, the initial charge-discharge curves of EG-MNPs-Al shown in Fig. 5b are significantly different from that of NFG, indicating that the accommodation of lithium into NFG and EG-MNPs-Al materials are different. The voltage plateau at about 0.80 V *vs.* Li/Li^+^ of the first discharge curve of EG-MNPs-Al, represents the generation of irreversible capacity, which can be attributed to the electrolyte decomposition and the formation of a solid electrolyte interface (SEI) film on the electrode surface^14^,^15^,^38^. Typically, the insertion/extraction curves of EG-MNPs-Al imply the presence of four stages corresponding to four different Li-storage sites, i.e., a voltage plateau lower than 0.25 V (*vs.* Li/Li^+^), monotonic sloping voltage curve between 0.25 and 0.8 V (*vs.* Li/Li^+^), an inclined plateau from 0.8 to 1.6 V (*vs.* Li/Li^+^), and the slope curve above 1.6 V (*vs.* Li/Li^+^). According to previous studies^31^,^39^,^40^, the low-voltage plateau (0.25 V) may be assigned to the formation of Li-Al alloys and the Li^+^ insertion/extraction in the pores or defects, whereas the monotonic profile between 0.25~0.8 V is associated with the intercalation of Li^+^ between graphite layers in EG-MNPs-Al. The sloping plateau from 0.8 to 1.6 V may be due to the interaction between Li^+^ and the hydrogen-terminated dangling bonds^40^. The slope curve above 1.6 V could be possibly attributed to the faradic capacitance either on the surface of graphite layers or on the edge planes^16^. It is clear that the theoretical capacity of graphite electrode is 372 mAh·g^−1^, which corresponds to the formation of Li-graphite intercalation compound (Li-GIC, i.e., LiC_6_). However, as shown in Fig. 5b, the charge/discharge capacity of EG-MNPs-Al is much higher than 372 mAh·g^−1^. This result suggests that LiC_6_ compound is not sufficient to explain such a high storage capacity. Therefore, other mechanisms such as the formation of Li-Al alloy for the storage of lithium ion species becomes possible. Figure 5c exhibits the CVs of 1^st^ and 50^th^ cycles at a scan rate of 0.1 mV·s^−1^. Two reduction peaks (at 0.28 V and 0.78 V) and one oxidation peak (at 1.56 V) are observed, which might be ascribed to the formation of Li_*x*_Al (*x* = 1~2.25) alloys based on the alloy phase diagrams^31^. The two reduction peaks present the dissolution and reduction of Al with lithium, and the oxide peak is associated with the conversion to a Li_*x*_Al alloy. Although the CVs could illustrate the formation of Li_*x*_Al alloy in this anode material, further systematic works would be needed in the future. Both anodic and cathodic peaks are positively shifted, which might be attributed to polarization of the electrode materials in the cycles. This anode material shows very stable repeatability after 50 cycles, signifying that the EG-MNPs-Al anode has a high reversible capacity and long cycling life. It was in agreement with the charge-discharge curves (Fig. 5b).

From above experimental results, it is apparent that Al nanoparticles embedded in EG are contributed to the specific capacity and cyclic performance. One aspect, the initial capacity of EG-MNPs-Al showed an improvement of 45% compared to EG, it could be attributed to the formation of Li_*x*_Al alloys. Another aspect, the existence of Al nanoparticles could improve the parallel thermal conductivity of EG, and the excellent thermal conductivity would show a positive influence on the cycling stability of the EG-MNPs-Al electrode by enhancing the heat transfer speed of LIBs system. Overall, the Al nanoparticles play an important role in improving the electrochemical performance of EG-MNPs-Al.

The cycling stability of the EG-MNPs-Al material was investigated at a current density of 100 mA·g^−1^ for 300 cycles, as shown in Fig. 5d. The initial cycle CE of EG-MNPs-Al was 74% during cycling, rather lower than that of NFG (Fig. 5a), and reached above 98% after 5 cycles. This phenomenon was similar to that of EG which exhibited a first cycle CE of 71% (Fig. 5e), indicating that such a first cycle CE of EG-MNPs-Al was related to the microstructure of the host EG. Compared to the NFG, EG-MNPs-Al and EG possessed more functional groups, pores or defects, so that a number of Li^+^ would be consumed and arrested by the functional groups or defects, even Al nanoparticles and thus become dead Li during the first charge process. Therefore, such a first cycle CE and irreversible capacity could be usually ascribed to the reaction between lithium ions and the surface functional groups and the formation of an SEI film in pores or defects derived from the oxidation/wrinkle^41^,^42^,^43^. The EG-MNPs-Al electrode delivered a specific capacity of 400 mAh·g^−1^ after 300 cycles with a high capacity retention of 84%. It demonstrated excellent cycling stability with a very low capacity decay rate of 0.054% per cycle from the 1^st^ cycle to the 300^th^ cycle. The high cycling performance delivered by the EG-MNPs-Al electrode is mainly ascribed to the unique structure and its excellent thermal conductivity. One aspect, the enlarged interlayer space and porous structure of EG-MNPs-Al can reduce the resistance of Li^+^ transport to make the insertion/extraction of Li^+^ easily to take place. Another aspect, the excellent thermal conductivity of EG-MNPs-Al can enhance the heat transfer speed so as to improve the cycling stability of the Li-ion battery. Figure 5f shows the comparison curves of the charge/discharge cycling stability for EG-MNPs-Al, EG, and NFG. For the EG-MNP-Al, the reversible capacity after 20 cycles was 460 mAh·g^−1^, and 96% retention of the initial capacity. However, the reversible capacity after 20 cycles was 258 and 280 mAh·g^−1^ for NFG and EG, respectively. The reversible retention capacity after 20 cycles was 81 and 85% for NFG and EG, respectively. The experimental results exhibit that the specific capacity of EG-MNPs-Al is much larger than that of EG and NFG, and also the fading capacity of EG and NFG is obviously faster than that of the EG-MNPs-Al. It can be inferred that the excellent thermal conductivity of EG-MNPs-Al mainly resulting from the presence of Al metal plays a key role in its cycling performance. During the long-term cycling process, the electrode materials may generate large amounts of heat and that need to be released immediately^10^. Otherwise, the large amounts of accumulated heat not only cause safety issues (e.g. battery explosion) but also speed up the capacity deterioration. So, the thermal property of electrode materials strongly affects the electrochemical performance for Li-ion batteries. It is very important to synthesize a candidate material having good thermal property to release the accumulated heat in battery system. Meanwhile, this heat-medium material also has good electrochemical performance as electrode material for Li-ion batteries. In this paper, the EG-MNPs-Al material synthesized by an oxidation-expansion process has excellent thermal conductivity to easily release the heat by the heat conduction style in battery system, and it also has good electrochemical performance. Therefore, it is favorable to improve the heat management and cycling stability of Li-ion batteries.

The rate capability of the EG-MNPs-Al electrode was evaluated at different current densities from 100 to 1000 mA·g^−1^ (Fig. 5g). The cell was firstly cycled at 100 mA·g^−1^ for 10 cycles, under which a high specific capacity of around 475 mAh·g^−1^ was obtained. When the current density increases from 100 to 500 mA·g^−1^, the available capacity keeps a stable value of ca. 350 mAh·g^−1^ for the EG-MNPs-Al, which is higher than the first reversible capacity of NFG at 100 mA·g^−1^ (Fig. 5a). The EG-MNPs-Al electrode could still deliver a reversible capacity of ca. 270 mAh·g^−1^ after 10 cycles even at a high current density of 1000 mA·g^−1^. When the current density is again reduced back to 100 mA·g^−1^, the capacity is recovered to about 450 mAh·g^−1^ with a low capacity decay. The results indicate that the EG-MNPs-Al electrode material possesses a good rate performance and cycling stability.

As a result, EG-MNPs-Al electrode material has superior thermal conductivity and excellent electrochemical performance as anode material for Li-ion batteries.

Although the detailed lithium insertion/extraction mechanism of EG-MNPs-Al is not clear, it was proposed that the reversible capacity of them varied significantly depending on the metal embedded network structure. The possible reasons are: the electronic structure of EG-MNPs-Al will be different from that of graphite; the metal embedded network structure of EG-MNPs-Al may cause additional sites for accommodation of lithium ions. Not only the regular sites of LiC_6_, but also the Li_*x*_Al alloying would be available because of both the structural and electronic changes in the EG-MNPs-Al materials.

**Figure 6:**
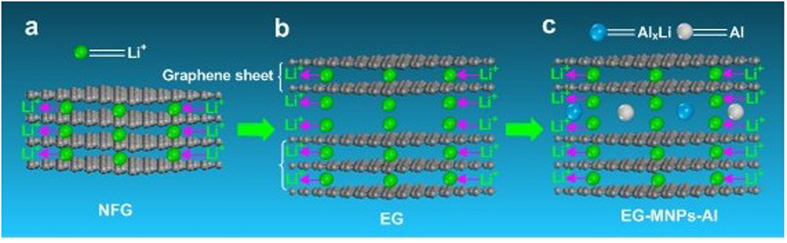
Insertion/extraction mechanisms. Schematic diagrams of the insertion/extraction mechanisms of Li+ in (**a**) NFG, (**b**) EG and (**c**) EG-MNPs-Al.

In order to further understand the high reversible capacity and good cyclic stability of the EG-MNPs-Al material, the possible intercalation mechanisms of Li^+^ in NFG, EG and EG-MNPs-Al are schematically depicted in Fig. 6. The interpretive schematic shows that the Li^+^ insertion/extraction mechanisms of NFG, EG and EG-MNPs-Al are different from each other. According to the references^16^,^43^, Li ions tend to be electrochemically absorbed on both sides of single-layer sheets that are arranged horizontally. The Li^+^ intercalated sites in graphite are predominantly interlayer space between the adjacent graphite layers due to the lamellar structure (Fig. 6a). However, the EG-MNPs-Al possesses a layer-by-layer structure with an enlarged interlayer lattice distance. It is more favorable to the electrochemical absorption of Li^+^ between the graphite layers, and can offer more Li^+^ insertion active sites^44^. Furthermore, the curled structure and disorder stacking of graphene sheets provide large amounts of pores or defects, resulting in a geometrical increase of Li^+^ intercalation numbers. Due to the enlarged interlayer space and the existence of graphene sheets, the unique structure of EG-MNPs-Al has many channels that can provide more effective Li^+^ insertion sites so that the diffusion of Li^+^ takes place easily. Thus, it effectively facilitates Li^+^ to reversibly insert into and extract from the electrode materials and thereby limits the formation of dead lithium. This reversible insertion/extraction is responsible for the excellent cycling stability of the electrode materials. Most importantly, the EG-MNPs-Al material possesses a particular hierarchical structure with metal particles embedded into the interspaces of EG, so that the alloying reaction between Al and Li may take place during the cycling (Fig. 6c). Owing to the formation of Li_*x*_Al alloy, the Li^+^ storage capability of EG-MNPs-Al can be enhanced, higher than that of EG (Fig. 6b). And this is also the most important reason why the EG-MNPs-Al material delivers a higher reversible capacity than the other EG reported by previous studies^17^,^18^,^19^. Therefore, the particular structure of the EG-MNPs-Al electrode material plays an important role in enhancing the large reversible capacity and cycling stability.

Furthermore, the above results demonstrate that the EG-MNPs-Al electrode material is effective in the thermal management system for Li-ion battery modules, and the presence of metal nanoparticles is beneficial for performance of batteries operating under certain conditions. In addition, the excellent thermal conductivity of electrode materials makes the temperature successfully regulated and uniformly distributed throughout the battery modules. The temperature control produced a significant improvement in battery cycle life, which is consistent with similar results reported elsewhere in the literature^45^.

## Discussion

In summary, EG-MNPs-Al electrode materials were successfully synthesized by the oxidation-expansion process using natural flake graphite as the raw material and Al compound as the expanding agent. The hierarchical structure of EG-MNPs-Al and wrinkled graphene sheets can provide more lithium insertion active sites and effectively facilitate the Li^+^ diffusion. The metal embedded network structure can enhance the parallel thermal conductivity and the formation of Li_*x*_Al alloy can improve the Li^+^ storage capability of EG-MNPs-Al. The parallel thermal conductivity of EG-MNPs-Al was up to 11.6 W·m^−1^·K^−1^ with a bulk density of 453 kg·m^−3^ at room temperature. Compared to EG, it was a 150% improvement mainly owing to the existence of Al metal. The first reversible capacity of EG-MNPs-Al material was 480 mAh·g^−1^ at a current density of 100 mA·g^−1^, and retained 84% capacity after 300 cycles, even at a high current density of 1000 mA·g^−1^, it could still deliver a reversible capacity of ca. 270 mAh·g^−1^ after 10 cycles. The excellent thermal conductivity of EG-MNPs was believed to be the primary factor to improve the cycling stability of Li-ion battery. Therefore, such flexible anode materials with outstanding electrochemical performance and thermal properties can be a potentially promising candidate for LIBs.

## Methods

### Material Synthesis

EG was prepared by a modified method based on the previous work^20^,^21^,^22^. To convert the pristine natural flake graphite (NFG) into an intercalated graphite, the flake graphite (98%) and KMnO_4_ (analytical pure grade, as the oxidizing agent) were mixed and saturated with acids consisting of concentrated sulfuric acid (98%) and nitric acid (65%) under magnetic stirring at 25 °C. After reaction for 30 min, the mixture was filtered and washed with deionized water till the pH level of the resulting solution reached 5, and then was dried at 80 °C. Furthermore, the resulting mixture was placed into an Al compound (such as AlCl_3_) gas atmosphere with the Al metal as the expanding agent. EG was then obtained by a rapid expansion and exfoliation of the expandable graphite after putting in a muffle furnace at 1000 °C for 10 s, denoted as EG-MNPs-Al. For comparison, the sample without the Al compound was also prepared as a control sample in the experiment. The specific characteristics of as-prepared samples are shown in Table 1.

### Characterization

The morphology and microstructure of the resulting samples were characterized using field-emission scanning electron microscopy (FESEM, JSM-6700F, JEOL), high resolution transmission electron microscopy (HRTEM, Tecnai G2 F30, FEI), X-ray diffraction with Cu K_α_ radiation (XRD, X’Pert PRO MPD, PANalystal, λ = 0.154 nm), and Raman spectroscopy (514 nm, LabRAM HR800, HORIBA JOBIN YVON). The chemical state of Al and surface elemental composition were determined by X-ray photoelectron spectroscopy (XPS) analysis using a K-Alpha spectrometer (Thermo Scientific) equipped with Mg-K_α_ X-ray radiation. Electrochemical measurements were carried out using a CHI 650D electrochemical workstation (Shanghai Chenhua Co., Ltd., China) with a coin cell at the scan rate of 0.1 mV·s^−1^. The thermal conductivity and diffusivity of the samples were characterized using Laser Flash Apparatus (LFA 447 Nanoflash, NETZSCH Scientific Instruments Trading (Shanghai) Ltd.) based on ASTM D5470 standard from room temperature to 300 °C. To analyze the thermal properties of the experimental samples, the diameter of 12.7 mm and the thickness of 0.1 mm thin films with walls parallel to the compressing direction were prepared by calendering molding. The thermal conductivity measurements show that the perpendicular thermal conductivities (relative to the direction of compression, 40~500 W·m^−1^·K^−1^) of the thin films are significantly higher than the thermal conductivities in the parallel direction, so that we only need to pay attention to the influence of parallel thermal properties on the battery performance in the following discussion.

### Electrochemical measurements

The electrochemical experiments were carried out using CR2025 coin-type cells. The working electrodes contained 84 wt.% active materials, 10 wt.% carbon black and 6 wt.% polyvinylidene fluoride (PVDF). The electrolyte was 1 M LiPF_6_ in solution of 1:1 volume ratio of ethylene carbonate (EC) and diethyl carbonate (DEC). Celgard 2300 film was used as separator and pure lithium metal foil was used as the counter electrode. The coin cells were assembled in an argon-filled glove box (H_2_O < 0.1 ppm, O_2_ < 0.1 ppm). Galvanostatic charge-discharge measurements were conducted in the voltage range from 0.01 to 3 V vs. Li/Li^+^ with a multi-channel battery analyzer (BTS-5V10mA, Shenzhen Neware Technology Co., Ltd., China) at room temperature.

## Additional Information

**How to cite this article**: Zhao, T. *et al*. Expanded graphite embedded with aluminum nanoparticles as superior thermal conductivity anodes for high-performance lithium-ion batteries. *Sci. Rep.* **6**, 33833; doi: 10.1038/srep33833 (2016).

## Figures and Tables

**Table 1:** The specific characteristics of EG and EG-MNPs-Al.

Samples	Al weight ratio (wt.%)*	Bulk density (*d*) (kg·m^−3^)	*d*_s_/*d*_s1_**
EG	0	396	1
EG-MNPs-Al-1	5	427	1.07
EG-MNPs-Al-2	10	453	1.14
EG-MNPs-Al-3	15	469	1.18

^*^wt.% = (m_2_−m_1_)/m_1_·100%, m_1_ represents the mass of the expandable graphite before Al metal intercalation, m_2_ represents the mass of EG-MNPs-Al.

^**^d_s_ represents the bulk density of samples, *d*_s1_ represents the bulk density of EG.
